# Num1 versus NuMA: insights from two functionally homologous proteins

**DOI:** 10.1007/s12551-018-0472-x

**Published:** 2018-11-06

**Authors:** Samuel R. Greenberg, Weimin Tan, Wei-Lih Lee

**Affiliations:** Department of Biological Sciences, Dartmouth College, Hanover, NH 03755 USA

**Keywords:** NuMA, Num1, Dynein, Spindle orientation

## Abstract

In both animals and fungi, spindle positioning is dependent upon pulling forces generated by cortically anchored dynein. In animals, cortical anchoring is accomplished by a ternary complex containing the dynein-binding protein NuMA and its cortical attachment machinery. The same function is accomplished by Num1 in budding yeast. While not homologous in primary sequence, NuMA and Num1 appear to share striking similarities in their mechanism of function. Here, we discuss evidence supporting that Num1 in fungi is a functional homolog of NuMA due to their similarity in domain organization and role in the generation of cortical pulling forces.

## Introduction

Proper orientation of the mitotic spindle is paramount to many fundamental processes in cell and developmental biology. In particular, orienting the mitotic spindle ultimately controls the outcome of asymmetric cell division, which is crucial for determining daughter cell location, size, and fate. From animals to fungi, pulling forces for orienting the spindle are generated by cytoplasmic dynein, a conserved microtubule ATPase. In many cases, dynein is anchored at the cell cortex and pulls on the spindle poles through the connecting astral microtubules (aMTs), thereby aligning the spindle along the axis of polarization (McNally 2013; Siller and Doe 2009). This mechanism requires dynein to interact with aMTs and its anchoring proteins at the cell membrane.

In many well-studied animal models of spindle orientation—such as *Drosophila* neuroblasts, *C. elegans* zygotes, and cultured human HeLa cells—dynein associates with the cortex via interaction with the NuMA family of proteins (Couwenbergs et al. 2007; Kotak et al. 2012; Nguyen-Ngoc et al. 2007; Okumura et al. 2018). NuMA interacts with the LGN family of proteins (Bowman et al. 2006; Du and Macara 2004; Siller et al. 2006), which in turn bind to the myristoylated heterotrimeric Gαi protein that is directly attached to the membrane. Additionally, in vertebrate skin basal progenitors and neuroepithelial progenitors, the same ternary complex NuMA-LGN-Gαi regulates asymmetric divisions and planar cell divisions, respectively, using dynein at the cortex to control spindle orientation (Peyre et al. 2011; Williams et al. 2011). In contrast, in budding yeast, one of the earliest model systems for spindle orientation (Eshel et al. 1993; Li et al. 1993), dynein associates with the cortex via interaction with the single component cortical anchoring protein Num1. BLAST searches identified clear homologs of Num1 in fungi but not in animals. Conversely, clear homologs of NuMA are found in animals but not in fungi.

**Fig. 1:**
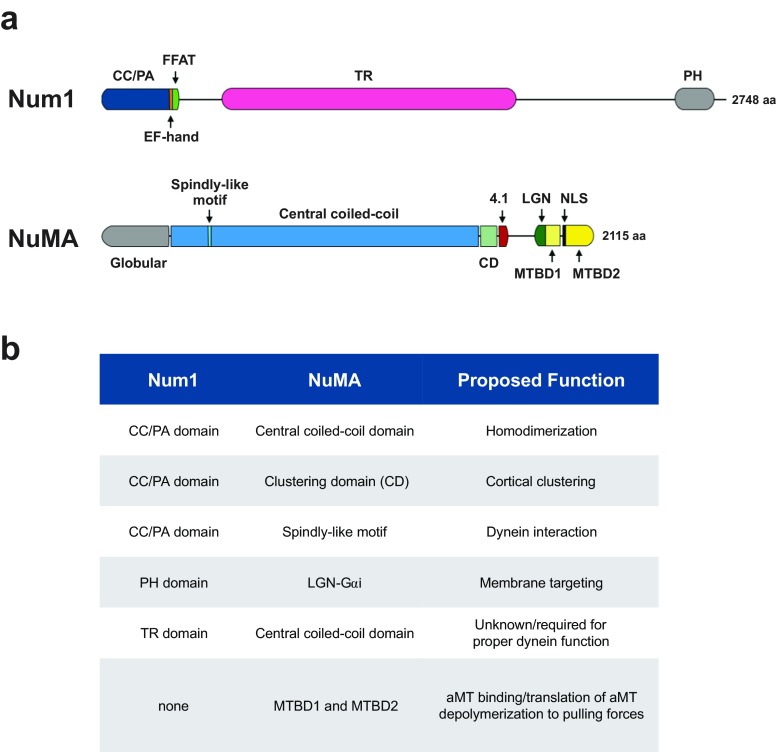
Num1 and NuMA share functionally homologous domain structures. **a** Diagrams of Num1 and NuMA. **b** Summary of domains in Num1 and NuMA that specify similar functions in dynein interaction, membrane targeting, and cortical clustering. All are part of the Num1 and NuMA proteins except LGN-Gαi, which is functionally homologous to the PH domain of Num1

However, a common feature between Num1 and NuMA is that both are large, multi-domain proteins (Fig. 1a). Num1 is a 313 kDa protein composed of a short N-terminal coiled-coil domain (aa 95–303), followed by a putative Ca^2+^-binding EF-hand (aa 303–316) overlapping with a putative ER-targeting FFAT motif (aa 306–330), a central TR domain containing thirteen 64-residue tandem repeats (aa 592–1776), and a C-terminal lipid-binding PH domain (aa 2563–2683) (Chao et al. 2014; Tang et al. 2012). In comparison, NuMA is a 238 kDa protein composed of a small N-terminal globular domain (aa 1–212), followed by a spindly-like motif (aa 417–422) located within an extended coiled-coil region (aa 213–1699), and a C-terminal portion containing a cluster-forming CD domain (aa 1700–1801), as well as interaction domains for 4.1 family proteins (aa 1788–1810), LGN (aa 1892–1924), microtubules (aa 1914–1985 and aa 2002–2115), and a nuclear localization signal sequence (aa 1988–2005) (Chang et al. 2017; Du et al. 2002; Gallini et al. 2016; Harborth et al. 1999; Haren and Merdes 2002; Kotak et al. 2012; Mattagajasingh et al. 1999; Okumura et al. 2018; Seldin et al. 2016). Although the domains are different between the two proteins, many of them appear to carry out homologous functions in terms of how they facilitate dynein interaction, activation, cluster formation, and membrane localization (Fig. 1b) (Okumura et al. 2018; Tang et al. 2012). Thus, it would be wise to pay close attention to both animal and fungal systems in order to gain insights into the conserved regulatory mechanisms governing cortically anchored dynein.

## Num1 and NuMA both dimerize and exhibit cortical clustering activity

Current evidence shows that Num1 and NuMA both form homodimers. Homodimerization of Num1 was established through co-immunoprecipitation, gel filtration, and equilibrium sedimentation analysis of recombinantly tagged constructs of Num1. Specifically, homodimerization is mediated by amino acids 95–303 of the N-terminal coiled-coil domain, also termed the CC or PA domain (Tang et al. 2012). A smaller fragment encompassing amino acids 97–294 also forms dimers (Ping et al. 2016). The same is accomplished by amino acids 213–1699 in the central rod–like coiled-coil domain of NuMA, established through chemical crosslinking studies, circular dichroism spectra, and electron microscopy of NuMA constructs purified from *E. coli* (Harborth et al. 1999; Harborth et al. 1995). In addition to forming homodimers, both NuMA and Num1 appear to cluster on the cell membrane, forming punctate cortical foci in vivo (Farkasovsky and Kuntzel 1995; Heil-Chapdelaine et al. 2000; Okumura et al. 2018), a behavior shown to be required for proper spindle pulling. NuMA clustering is facilitated by amino acids E1768-P1777 found in the C-terminal CD domain (Okumura et al. 2018), whereas Num1 clustering is dependent upon E191 and K192 residues found in the N-terminal CC/PA domain (Tang et al. 2012). Remarkably, both NuMA and Num1 patches are independent of filamentous actin (Berends et al. 2013; Heil-Chapdelaine et al. 2000; Okumura et al. 2018; Omer et al. 2018), suggesting that the cortical dynein attachment site may have similar architecture in fungi and animals. Num1 harboring mutations in the E191 and K192 residues could not assemble bright patches and exhibited a strong spindle misorientation phenotype (Tang et al. 2012), indicating defective dynein pathway function. Similarly, NuMA harboring mutations in the CD domain failed to form punctate foci and could not fully displace the spindle (Okumura et al. 2018). Patch formation is therefore critical for the activity of both anchors, but whether dimerization is important for patch formation and spindle pulling has yet to be established.

## Num1 and NuMA share analogous domains for dynein anchoring and membrane targeting

Both cortical anchors have an N-terminal domain that recruits and anchors dynein. Tang et al. (2012) used bimolecular fluorescence complementation and pull-down assays to show that dynein binds to the N-terminal CC/PA domain of Num1. Okumura et al. (2018) mapped the dynein-binding site in NuMA to the spindly-like motif sequence (aa 417–422) located near the N-terminal side of the central rod–like coiled-coil domain. Mutations in the spindly-like motif made NuMA unable to recruit dynein (Okumura et al. 2018), suggesting that this motif is functionally homologous to the Num1 CC/PA domain. Interestingly, Num1 constructs containing only the CC/PA domain fused to a membrane-targeting motif were able to move the spindle (Tang et al. 2012), while NuMA constructs containing only the spindly-like motif fused to a light-induced membrane–targeting anchor were unable to do the same. This implies that, while the CC/PA domain and the spindly-like motif are sufficient to interact with dynein, only the CC/PA domain is able to also serve as an activator of dynein for spindle pulling.

In addition to their similar dynein-anchoring domains, both Num1 and NuMA have functionally homologous cortical attachment machinery. Num1 is targeted to the cortex via its C-terminal pleckstrin homology (PH) domain, which binds the membrane phospholipid phosphatidylinositol 4,5-bisphosphate (PIP_2_) with high affinity and specificity (Yu et al. 2004), and serves to localize Num1 to the cell membrane (Tang et al. 2009). However, the PH domain is not sufficient for cluster formation as isolated PH domain does not oligomerize to form bright puncta at the cell cortex (Tang et al. 2009). Instead, fusions of the CC/PA domain and the PH domain were found to be the minimal constructs capable of assembling into bright cortical patches (Lackner et al. 2013; Ping et al. 2016; Tang et al. 2012), indicating that the CC/PA domain is also required for cluster formation. Interestingly, the only process dependent on the PH domain appears to be membrane targeting, since its deletion can be rescued by addition of a generic membrane–targeting tag (Schmit et al. 2018; Tang et al. 2012, 2009). Thus, the PH domain is dispensable for cluster formation and spindle pulling once Num1 reaches the cell cortex.

NuMA is also targeted to the membrane via machinery dispensable for spindle pulling. As a member of the ternary complex, NuMA is targeted to the linker protein LGN, which in turn is targeted to the membrane-bound heterotrimeric Gαi protein. Thus, LGN-Gαi is functionally homologous to the PH domain of Num1, since they serve to anchor their respective dynein-binding domain to the cell cortex. In striking similarity to Num1, ectopic optogenetic targeting of NuMA to the cell membrane in the absence of LGN-Gαi was found to induce high spindle pulling force (Fielmich et al. 2018; Okumura et al. 2018). This would suggest that the cortical attachment machinery (PH domain for Num1; LGN-Gαi for NuMA) is dispensable for cortical force generation.

While the cortical attachment machinery is not necessary for force production, it might be involved in dynein regulation. In budding yeast, Omer et al. (2018) demonstrated that the spatial distribution of Num1 along the cell cortex exerts fundamental regulation on the mechanism of dynein pulling. The signaling pathway necessary for Num1 to communicate its localization into regulation of dynein has not been established, but it is conceivable that the PH domain of Num1 is involved. Gαi has also been shown to be a key target for upstream regulators of dynein in ternary complex–containing cells (Ananthanarayanan 2016; Couwenbergs et al. 2007; Fielmich et al. 2018). Thus, although the membrane attachment machinery of NuMA and Num1 are dispensable for baseline function, they should not be ignored as sites for the input of regulation. The complexity of the heterodimeric attachment species (i.e., LGN and Gαi) in the ternary complex compared to the relatively simple attachment domain in Num1 likely reflects the greater volume of regulation required by dynein in complex metazoans.

Num1 and NuMA share a central domain of important but unidentified purpose. In Num1, a central region containing thirteen 64-residue tandem repeats (TR) links the N- and C-terminal functional domains. The role of this domain has not been identified, and its deletion neither disrupts Num1 clustering nor increases the proportion of binucleated cells, indicating normal dynein pathway function (Tang et al. 2012). These data would suggest that the TR domain is irrelevant for spindle pulling, but a more sensitive assay found that cells missing the TR domain had a lower percentage of spindles crossing the bud neck and a reduced spindle penetration distance when crossing occurred (Tang et al. 2012). This suggests that the TR domain is pertinent to Num1’s activity as a cortical anchor, but its role has yet to be identified. Similarly, NuMA contains a large region of unspecified purpose between its N- and C-terminal domains. Though the function of this central coiled–coil domain has not been determined, constructs of NuMA containing only the N- and C-terminal functional domains were unable to induce high spindle pulling forces (Okumura et al. 2018). Thus, the central coiled-coil of NuMA also plays a role in the generation of cortical pulling forces, similar to the central TR domain of Num1.

## The presence of MTBDs in NuMA but not Num1 is reflective of their differing mechanisms for the initiation of spindle pulling

A significant difference between NuMA and Num1 is the unique presence of two microtubule-binding domains (MTBD1 and MTBD2) in NuMA. These domains, located near the C-terminal region of NuMA, are required for generation of spindle pulling forces (Okumura et al. 2018). However, their specific role in this process is incompletely understood. It was proposed that the MTBDs may either function to couple shrinking microtubule ends to the cortex to generate pulling forces (Okumura et al. 2018), or facilitate the capture of aMTs by dynein (Serra-Marques and Dumont 2018). To evaluate the significance of the NuMA MTBDs and their apparent lack of homology in Num1, an understanding is required of the differing processes of aMT/dynein/cortical anchor complex assembly in Num1 and NuMA containing cells.

For spindle pulling, dynein must be bound both to its cortical anchor and to an aMT. This is true in both Num1 and NuMA containing cells, but the order in which these components associate is critically different between the two types. In the current yeast model, dynein freely exists in the cytoplasm until it is recruited to the plus end of an aMT. Once the plus end contacts Num1, dynein offloads to the cortex and begins to exert pulling forces on the aMT (Ananthanarayanan et al. 2013; Lee et al. 2005, 2003; Markus and Lee 2011; Sheeman et al. 2003). In contrast, dynein in NuMA containing cells is recruited directly from the cytoplasm to NuMA’s spindly-like motif, and there it waits to “capture” an aMT and pull to orient the spindle (Collins et al. 2012; Kiyomitsu and Cheeseman 2012). The key difference between these pathways is that dynein bound to NuMA must “capture” aMTs whereas dynein bound to Num1 does not. Thus, the idea posited by Serra-Marques and Dumont (2018) that the NuMA MTBDs are required for stabilizing the aMT/dynein interaction is reconciled with the absence of an MTBD in Num1, since dynein on Num1 is usually associated with an aMT and likely needs no further stabilization of this interaction. This would suggest that there is no homolog of the NuMA MTBDs in the Num1 pathway, but recent data has indicated that the MTBD has a separate function related to that of dynactin in yeast.

Seldin et al. (2016) and Okumura et al. (2018) suggested that the MTBDs of NuMA co-generate cortical pulling forces alongside dynein. Okumura et al. (2018) showed that a NuMA fragment containing MTBD1 accumulated at the plus ends of aMTs and remained associated as they depolymerized. This indicates that the MTBDs may harness the energy of the depolymerizing aMT to generate pulling forces parallel to those produced by dynein (Okumura et al. 2018). The NuMA MTBDs have no equivalent domain in Num1, but they may bear functional homology to a domain in the p150^glued^ subunit of dynactin. The budding yeast homolog of dynactin p150^glued^ subunit, Nip100, contains a CAP-Gly domain. Omer et al. (2018) recently showed that deletion of the CAP-Gly domain resulted in a significant decrease in the duration of aMT-cortical dynein interactions during end-on aMT pulling events. This suggests that CAP-Gly is involved in tethering the depolymerizing aMT to the cell cortex, in order to translate the energy of the depolymerizing aMT into spindle pulling forces (Omer et al. 2018). This data thus implicates the CAP-Gly domain of yeast dynactin as the functional homolog of the NuMA MTBDs.

## Num1 organelle association and future research

In budding yeast, Num1 is known to associate with both mitochondria and cortical ER (Cerveny et al. 2007; Chao et al. 2014; Hammermeister et al. 2010; Klecker et al. 2013; Kraft and Lackner 2017; Lackner et al. 2013; Omer et al. 2018; Tang et al. 2012). Kraft and Lackner (2017) showed that inhibiting mitochondrial migration into the bud using temperature sensitive mutants resulted in significantly reduced Num1 patch formation. It has also been observed in several studies that Num1 anchors the mitochondria to the cell cortex via its CC/PA domain (Lackner et al. 2013; Ping et al. 2016; Tang et al. 2012). This suggests that Num1 patch assembly is dependent on mitochondria, and that patches then serve to anchor both dynein and the mitochondria to the cell cortex. Interestingly, Num1 appears to also associate with the cortical ER, bringing dynein, mitochondria, and ER into close proximity near the plasma membrane. Deletion of the cortical ER tethering proteins Scs2 and Scs22 was found to almost completely eliminate Num1 localization to the lateral cortex, while enhancing localization to the bud tip (Chao et al. 2014; Omer et al. 2018). This indicates that Num1 is likely involved in ER tethering and may take part in ER-dependent regulation of dynein. To our knowledge, the ternary complex has not been observed to associate with any organelles other than the ER, which has been implicated in Gα regulation in *C. elegans* embryos (Berends et al. 2013). Investigating whether the ER and/or other organelles substantively interact with the ternary complex in *C. elegans* embryo or other ternary complex containing cells is thus a next step in the study of animal dynein regulation.
